# Dopamine synthesis capacity in patients with remitted psychosis: correlates and one year outcome on relapse

**DOI:** 10.1007/s00406-025-02185-8

**Published:** 2026-01-06

**Authors:** Christy Lai Ming Hui, Charlie Cheuk Lam Wong, Nicole Cai Lin Yang, Eunice Man Hei Choi, Priscilla Wing Man Hui, Eddie Chi Yuen Lui, Junchen Tiffany Tao, Elise Chun Ning Ho, Yi Nam Suen, Sirong Chen, Eric Yim Lung Leung, Garrett Chi Lai Ho, Gladys Goh Lo, Charles Wai Hong Chan, Wai Song Yeung, Lap Tak Poon, Simon Sai Yu Lui, Edwin Ho Ming Lee, Sherry Kit Wa Chan, Wing Chung Chang, Eric Yu Hai Chen

**Affiliations:** 1 Department of Psychiatry, School of Clinical Medicine, LKS Faculty of Medicine, The University of Hong Kong, Hong Kong, China; 2 School of Nursing, LKS Faculty of Medicine, The University of Hong Kong, Hong Kong, China; 3 Department of Nuclear Medicine & Positron Emission Tomography, Hong Kong Sanatorium & Hospital, Hong Kong, China; 4 Department of Diagnostic & Interventional Radiology, Hong Kong Sanatorium & Hospital, Hong Kong, China; 5 Department of Psychiatry, Pamela Youde Nethersole Eastern Hospital, Hong Kong, China; 6 Department of Psychiatry, United Christian Hospital, Hong Kong, China; 7 Department of Psychiatry, Castle Peak Hospital, Hong Kong, China; 8 The National Centre of Excellence in Youth Mental Health, Orygen, Australia; 9 Centre for Youth Mental Health, University of Melbourne, Melbourne, Australia

**Keywords:** Positron emission tomography, Dopamine, Relapse, Antipsychotics, Psychosis

## Abstract

**Background:**

Biological predictors of relapse in psychosis remain underexplored. We examined whether dopamine synthesis capacity (DSC) predicts psychotic relapse one year after remission, and its associations with baseline clinical and cognitive factors.

**Study Resign:**

In a prospective one-year follow-up study, forty-seven patients with remitted psychosis and fifteen age- and sex-matched healthy controls underwent positron emission tomography/computed tomography (PET/CT) scanning at baseline, with DSC indexed by mean standardized uptake value ratios (SUVr). Relapse was assessed monthly over one year.

**Study Results:**

SUVr measures did not differ between patients and controls. Within patients, a higher putamen-to-cerebellum SUVr ratio at baseline was associated with male sex (*p* = 0.019), unmarried status (*p* = 0.044), more premorbid schizoid-schizotypal traits (*p* = 0.015), and greater general psychopathology (*p* = 0.010); only the latter remained significant after correction for multiple comparisons. Higher caudate-to-cerebellum SUVr was also linked to schizoid-schizotypal traits (*p* = 0.007), while higher putamen-to-caudate SUVr correlated with better insight (*p* = 0.017). At one year, relapsers showed lower putamen-to-caudate SUVr (*p* < 0.001), were more often immigrants (*p* = 0.002), had poorer insight into antipsychotic benefits (*p* = 0.003), and were more likely to have discontinued medication at baseline (*p* = 0.029). Penalized logistic regressions confirmed that lower baseline putamen-to-caudate SUVr predicted relapse independently of medication status at both baseline (*p* = 0.037) and follow-up (*p* = 0.044).

**Conclusions:**

Baseline reductions in striatal DSC predicted relapse regardless of medication, supporting its role as a marker of dopaminergic vulnerability.

**Supplementary Information:**

The online version contains supplementary material available at https://doi.org/10.1007/s00406-025-02185-8.

## Introduction

### Dopamine synthesis capacity (DSC) as an important state-marker in psychotic disorders

Schizophrenia is characterized by high relapse rates, particularly following medication discontinuation [1]. Even with continued treatment, up to 41% of patients relapse within a year after achieving remission from their first episode of psychosis (FEP) [2]. The dopamine hypothesis posits that schizophrenia involves dysregulated presynaptic dopaminergic function, with elevated dopamine synthesis capacity (DSC) consistently observed in patients with schizophrenia [3, 4], in individuals at clinical high risk [5], and in association with symptom severity [6].

### DSC as a predictor of relapse

Relapse prevention in psychosis depends on identifying clinical, cognitive, functional, and lifestyle risk factors [7, 8]. To date, only one study has investigated striatal DSC as a predictor of relapse, reporting that higher DSC before antipsychotic tapering predicted a shorter time to relapse within 12 weeks of full discontinuation, suggesting impaired dopamine autoregulation as a mechanism of relapse vulnerability following remission from first-episode psychosis [9]. However, its predictive value in naturalistic, non-experimental settings remains unknown.

Moreover, findings on DSC and symptom severity are inconsistent. Some studies report that higher DSC is associated with greater persistence of positive symptoms, whereas associations with negative symptoms are less consistent (*n* = 23) [10]. In a study including both schizophrenia and bipolar patients, a positive relationship between positive symptom severity and DSC was observed (*n* = 32), but this association was non-significant when analyses were restricted to schizophrenia alone (*n* = 16) [11]. Similarly, a study of patients with first-episode psychotic disorders found no association between DSC and symptom severity (*n* = 35) [12]. These inconsistencies likely reflect small sample sizes and heterogeneity across study populations.

### DSC measurement in psychosis studies

Notably, the measurement of DSC remains non-standardized in the literature. ^18^F-DOPA studies quantify presynaptic dopaminergic function using kinetic modeling (K influx) and standardized uptake value ratios (SUVr), with the former being most common [4, 9–11]. SUVr provides good test-retest reliability, with preliminary evidence indicating its cost-effectiveness in identifying treatment resistance in schizophrenia [13]. While SUVr provides a practical method for assessing metabolic activity, it is susceptible to various biological, methodological, and technological factors [14].

Additionally, only a few studies have examined striatal subregions due to limitations in scanner resolution. Meta-analytic evidence indicates elevated DSC in the putamen (*d* = 0.51) but not the caudate in schizophrenia [4]. Nonetheless, these analyses remain exploratory due to the limited evidence available. Given this, and to minimize susceptibility to external noise, we also used the caudate as a reference region for the putamen, providing a potentially more reliable estimate of putaminal DSC than conventional cerebellar referencing.

### Objectives

This study aimed to:

1. Compare DSC (SUVr indices) between patients in remission from psychosis and healthy controls;
2. Examine baseline demographic, clinical, and medication-related factors associated with DSC;
3. Test whether baseline putaminal DSC predicts relapse over one year in remitted psychosis.

## Methods and materials

### Subjects and study settings

Between June 2018 and August 2019, 47 participants were recruited from the Early Assessment Services for Young People at Early Psychosis (EASY). EASY provides specialized psychiatric care for young individuals with FEP during the first three years after onset, with extended follow-up when necessary, covering approximately seven million people in Hong Kong. Eligible participants, identified by attending clinicians, met the following criteria: (1) age 18 to 55 years, (2) diagnosis of schizophrenia or other non-affective psychoses (i.e., schizophreniform disorder, schizoaffective disorder, brief psychotic disorder, psychotic disorder not otherwise specified, and delusional disorder) according to the Diagnostic and Statistical Manual for Mental Disorders—Fifth Edition [15], and (3) the ability to understand verbal and written Chinese.

Exclusion criteria included intellectual disability, significant head trauma or neurological disorder, endocrine or cardiovascular disease, current or past illicit drug use, substance-induced psychosis, daily alcohol consumption over 5 standard units, metallic implants, claustrophobia, pregnancy or lactation, and comorbid psychiatric conditions.

Patients were required to be in remission for at least six months before study entry. Remission was defined using Andreasen’s criteria [16], requiring PANSS ratings of mild or less (≤ 3) on items P1, G9, P3, P2, N5, N1, N4, and N6. Participants were then followed prospectively for one year.

Additionally, we recruited 15 healthy controls aged 18 to 55 years with sufficient understanding of verbal and written Chinese. They did not meet any of the exclusion criteria: no history of antipsychotic use and no family history of schizophrenia. They were recruited via word-of-mouth and university email notifications.

Written informed consent was obtained from all the subjects. The study was conducted in accordance with Good Clinical Practice and the Declaration of Helsinki.

### Study procedures

This prospective study followed up 47 patients with remitted psychosis for one year. At study entry, all patients were assessed using positron emission tomography/computed tomography (PET/CT), basic demographics, symptoms, functioning, and cognitive function. A prospective monthly follow-up of relapse outcomes was performed for up to one year. For healthy controls, basic demographics, PET/CT imaging, and cognitive function were measured using a one-off assessment on the scan date. No follow-up was carried out for these healthy controls. All assessments were conducted in person by trained research assistants or postgraduate students.

### Measures

#### DSC

All participants underwent PET/CT brain imaging at the Hong Kong Sanatorium and Hospital Department of Nuclear Medicine and PET. The brain was scanned using an integrated in-line PET/CT (Biograph 40) in 3D mode. Data acquisition began with CT (with no contrast) at 130 kV, 110–115 mA, 2-mm pitch, and 1-second rotation, followed by PET of 148 axial image planes simultaneously at a 21.6 cm axial field of view. Before the scan, patients received an intravenous injection of ~ 370MBq ^18^F-DOPA. Scanning began at 30, 60, and 90 min after injection, each with a 10-minute emission acquisition. DSC levels were measured using the mean SUVr. SUVr of striatal (putamen and caudate) regions to cerebellar hemisphere, and the putamen to caudate SUVr were generated.

#### Clinical and functional assessments

Medication data (e.g., antipsychotic dosage and class) were extracted from the hospital’s Clinical Management System at both baseline and one-year follow-up.

Premorbid dysfunction was assessed using the Premorbid Schizoid and Schizotypal Traits (PSST) scale. Psychotic symptoms were assessed with the PANSS [17], and the Clinical Global Impression (CGI) Scale [18]. Depressive symptoms were assessed with the Calgary Depression Scale for Schizophrenia (CDSS) [19]. Insight was rated using the Scale to Assess the Unawareness of Mental Disorders (SUMD) [20], and medication side-effects were rated with the Udvalg for Kliniske Undersøgelser (UKU) [21]. The Social and Occupational Functioning Assessment Scale (SOFAS) was used to measure functioning [22].

#### Relapse outcome

Relapse was prospectively measured monthly over one year and was operationally defined as a score above the threshold for any one of the following five psychotic symptoms on the PANSS: delusions ≥ 3, conceptual disorganization ≥ 4, hallucinations ≥ 3, suspiciousness ≥ 5, or unusual thought content ≥ 4. Additional requirements included a score of ≥ 3 on the CGI Severity of Illness subscale and ≥ 5 on the CGI Improvement subscale. Patients were considered relapsed if they exceeded the threshold on at least one of five PANSS items and both CGI global scores for at least one week [2]. This definition allows for the detection of symptom exacerbation, enabling identification of mild relapses before severe deterioration or hospitalization.

Patients were assessed at study entry (baseline) and then monthly for one year to detect any relapse. Patients completed the study either upon relapse or upon completion of the follow-up, whichever occurred earlier.

### Data analysis

#### PET/CT data analysis

The regions of interest, including the right and left putamen, the right and left caudate, cerebellar hemispheres, and the occipital lobe, were automatically placed on three sets of ^18^F-DOPA PET using MIMNeuro4.2. The mean SUVr of putamen-to-cerebellum, caudate-to-cerebellum, and putamen-to-caudate were calculated for ^18^F-DOPA PET images at 60 min.

#### Statistical analysis

IBM SPSS Statistics 28.0 was used for primary data analyses. Demographic characteristics and SUVr measures were compared between patients (*n* = 47) and healthy controls (*n* = 15) using independent t-tests and Chi-square tests. Pearson’s correlations and one-way ANOVAs were conducted to examine associations between baseline demographic, medication, and clinical variables and the three SUVr indices (putamen-to-cerebellum, caudate-to-cerebellum, and putamen-to-caudate).

SUVr values, along with demographic and clinical variables, were compared between relapsers and non-relapsers using independent *t*-tests or Mann-Whitney *U* tests, as appropriate, to identify potential predictors of relapse. To control for type I error, the Benjamini–Hochberg false discovery rate (FDR; *q* = 0.10) was applied to the analyses of baseline associations and SUVr indices, as well as factors associated with relapse.

Given that medication use is a well-established predictor of relapse [2], significant univariate SUVr predictors were further examined using penalized logistic regression models to assess their independent association with relapse at one year. These multivariate models included the significant SUVr index and medication status (on/off) at baseline or follow-up as predictors, with relapse at one year as the dependent variable. Penalized logistic regressions were conducted in R using the logistf package (Firth correction) [23].

## Results

### Sample characteristics and DSC

The sample included 62 participants, including 47 patients with remitted psychosis and 15 healthy controls (Table 1). Among the 47 patients, 48.9% (*n* = 23) were male, 55.3% (*n* = 26) were diagnosed with schizophrenia, and the average age was 34.2 years. Six patients dropped out of the study. The average follow-up time for all patients was 332 days (SD = 84 days). Among relapsers (*n* = 5), the mean follow-up time was 229 days (SD = 125 days), and among non-relapsers (*n* = 42), the mean follow-up time was 344 days (SD = 70 days). The two groups were comparable in age, sex, and employment status. The patients and healthy controls did not differ in any of the DSC measures.

**Table 1:** Characteristics of patients with remitted psychosis and healthy participants at baseline.

Baseline variables^a, b^		Patients (*n* = 47)	Healthy participants (*n* = 15)		Comparisons *p*	
**Demographics**						
Male,* n* (%)		23 (48.9)	7 (46.7)		0.878	
Age		34.2 (9.9)	33.7 (9.8)		0.865	
Education, in years		13.6 (3.6)	15.4 (3.4)		0.079	
Employed, *n* (%)		33 (70.2)	12 (80.0)		0.459	
Married, *n* (%)		16 (34.0)	4 (26.7)		0.595	
Born in Hong Kong,* n* (%)		36 (76.6)	–		–	
SZ diagnosis, *n* (%)		26 (55.3)	–		–	
DUI, in days, median (IQR)		1783 (782–3862)	–		–	
DUP, in days, median (IQR)		40 (9–122)	–		–	
Antipsychotics dosage (CPZe)		226.7 (611.2)	–		–	
Medication type						
FGA		2 (4.3)	–		–	
SGA		36 (76.6)	–		–	
Mixed		1 (2.1)	–		–	
Off medication		8 (17.0)	–		–	
**Dopamine synthesis capacities**						
SUVr Putamen-to-Cerebellum		2.5 (0.23)	2.6 (0.22)		0.140	
SUVr Caudate-to-Cerebellum		2.0 (0.18)	2.0 (0.21)		0.622	
SUVr Putamen-to-Caudate		1.3 (0.08)	1.3 (0.07)		0.171	
**Clinical and functioning**						
PANSS						
Positive		7.2 (0.8)	–		–	
Negative		9.7 (4.4)	–		–	
General psychopathology		19.8 (3.6)	–		–	
CDSS		1.6 (2.3)	–		–	
SOFAS		66.5 (9.3)	–		–	

*n* = frequency, *SZ* = schizophrenia, *DUI = *duration of untreated illness, *DUP = *duration of untreated psychosis, *IQR* = interquartile range, *SUVr = * standardized update value ratio, *PANSS = *Positive and Negative Syndrome Scale, *CDSS = *Calgary Depression Scale for Schizophrenia, *SOFAS* = Social and Occupational Functioning Scale.

^a^ Unless stated otherwise, values represent means and standard deviations (in brackets).

^b^ There were no missing data in healthy controls, but missing data in patients were as follows: CDSS (*n* = 46) and SOFAS (*n* = 46).

### Baseline factors associated with DSC in patients

Between-group comparisons across diagnoses (i.e., schizophrenia [*n* = 26] and non-schizophrenia [*n* = 21]) suggested no statistically significant differences in all DSC measures. Higher DSC was associated with male patients, unmarried status, having greater impairment in premorbid schizoid and schizotypal traits, better insight, and more severe general psychopathology (Table 2).

**Table 2:** Baseline correlates of DSC in patients with remitted psychosis.

	SUVr					
	Putamen-to-Cerebellum		Caudate-to-Cerebellum		Putamen-to-Caudate	
	*r / M (SD)*	*p*	*r / M (SD)*	*p*	*r / M (SD)*	*p*
**Demographics**						
Age	− .15	0.336	− .11	0.449	− .06	0.712
Sex						
Male (n = 23)	**2.60 (0.23)**	**0.019**	2.06 (0.19)	0.088	1.27 (0.09)	0.375
Female (*n* = 24)	**2.44 (0.21)**		1.97 (0.16)		1.24 (0.08)	
Marital status						
Married (*n* = 16)	**2.43 (0.18)**	**0.044**	1.97 (0.16)	0.288	1.23 (0.06)	0.186
Others (*n* = 31)	**2.57 (0.24)**		2.03 (0.18)		1.25 (0.08)	
**Medication taken at scan**						
Antipsychotic dose (mg/day)^a^	.11	0.465	.11	0.461	0.0010	0.995
Medication discontinuation						
On-medication (*n* = 39)	2.52 (0.23)	0.937	2.01 (0.18)	0.915	1.25 (0.09)	0.942
Off-medication (*n* = 8)	2.52 (0.22)		2.02 (0.17)		1.25 (0.08)	
Medication class						
FGA (*n* = 2)	2.29 (0.05)	0.081	1.85 (0.01)	0.120	1.24 (0.02)	0.986
SGA (*n* = 36)	2.54 (0.22)		2.03 (0.18)		1.26 (0.09)	
FGA-SGA mix (*n* = 1)	2.06 (N/A)		1.67 (N/A)		1.23 (N/A)	
Off-medication (*n* = 8)	2.52 (0.22)		2.02 (0.17)		1.25 (0.08)	
Side effects						
UKU number of items	-0.0041	0.978	− .24	0.110	.27	0.069
**Clinical variables**						
PANSS						
Positive symptoms	− .08	0.616	.05	0.721	− .16	0.276
Negative symptoms	.08	0.617	− .11	0.452	.24	0.097
General psychopathology	**.37**	0.010*	.18	0.220	.25	0.093
CDSS	.01	0.954	.14	0.353	− .17	0.254
PSST	**.38**	**0.015**	**.42**	**0.007**	.05	0.739
SUMD						
Existence of mental illness	− .09	0.535	.19	0.212	**− .35**	**0.017**
Consequences of mental illness	.12	0.411	.24	0.111	− .14	0.365
Benefits of antipsychotics	− .15	0.352	.07	0.690	− .29	0.073

*SUVr =* standardized uptake value ratio, *r* = Pearson’s correlation coefficient, *M* = mean, *SD* = standard deviation, *n =* frequency, *FGA* = first generation antipsychotic, *SGA =* second generation antipsychotic, *UKU* = Udvalg for Kliniske Undersøgelser, *PANSS* = Positive and Negative Syndrome Scale, *CDSS* = Calgary Depression Scale for Schizophrenia , *PSST* = assessment of Premorbid Schizoid-Schizotypal Traits, *SUMD* = Scale to Assess Unawareness of Mental Disorder (lower score indicates better insight).

^a^ The mean daily dose of each antipsychotic was converted to an equivalent dose of chlorpromazine.

* Remained significant after multiple comparison correction.

Specifically, significantly elevated SUVr levels were observed in the putamen-to-cerebellum ratio among male individuals (*p* = 0.019) and unmarried patients (*p* = 0.044). The increased SUVr in the putamen-to-cerebellum was associated with more severe general psychopathology symptoms, as indicated by the PANSS scores (*p* = 0.010). Furthermore, both heightened SUVr ratios in the putamen-to-cerebellum (*p* = 0.015) and caudate-to-cerebellum (*p* = 0.007) were correlated with elevated PSST scores. Additionally, a higher putamen-to-caudate SUVr ratio was found in patients demonstrating better insight into the presence of mental illness, as measured by the SUMD scores (*p* = 0.017). However, only the heightened putamen-to-cerebellum ratio on general psychopathology remained significant after FDR correction.

### Predictors for psychotic relapse at one year

Among the 47 remitted patients, five (10.6%) experienced psychotic relapse during the one-year follow-up. All relapsers were females between the ages of 20 and 53. Three of them had schizophrenia, while the remaining two had other types of psychosis. Only two of the relapsing individuals were hospitalized during relapse episodes. Although two relapsers did not complete the SOFAS assessment at the time of relapse, the available data on functioning consistently indicated that relapsers exhibited lower functioning levels at relapse (**Supplementary 1**).

Relapsers displayed a significantly lower SUVr putamen-to-caudate (*p* < 0.001) than non-relapsers (Table 3). Beyond SUVr measures, relapsers are likely not to have been born in Hong Kong (*p* = 0.002), discontinued medication at baseline (*p* = 0.029), and have more impaired awareness towards the benefits of antipsychotics (*p* = 0.003). All associations remained significant after FDR correction.

**Table 3:** Predictors of relapse among patients in remission (n = 47).

Baseline predictors	Non- relapsers (*n* = 42)^a^	Relapsers (*n* = 5)	Statistics		Effect size
	M (SD) / Median (IQR)		t/X_2_/Z	*p*	d / *r*
SUVr putamen-to-cerebellum	2.54 (0.23)	2.36 (0.13)	1.63	0.110	0.77
SUVr caudate-to-cerebellum	2.01 (0.19)	1.99 (0.11)	0.25	0.801	0.12
SUVr putamen-to-caudate	1.26 (0.09)	1.19 (0.03)	4.27	< 0.001*	0.92
Immigrant status, *n* (%)	7 (16.67)	4 (80.00)	10.00	0.002*	0.46
Married, *n* (%)	14 (33.33)	2 (40.00)	0.09	0.776	0.04
SZ diagnosis, *n* (%)	24 (57.14)	2 (40.00)	0.53	0.466	0.11
DUP, in days (*n* = 46)	40.00 (11.50-122.50)	53.00 (8.00-896.50)	-0.07	0.944	0.01
Medication discontinuation, *n* (%)	5 (11.9)	3 (60.0)	N/A^b^	0.029*	0.40
CDSS	1.49 (2.27)	2.40 (2.88)	-0.83	0.414	-0.39
SUMD					
Existence of mental illness	1.10 (0.37)	1.40 (0.89)	-0.75	0.494	-0.68
Consequences of mental illness	1.20 (0.46)	1.40 (0.89)	-0.84	0.405	-0.40
Benefits of antipsychotics	1.23 (0.49)	2.00 (0.71)	-3.12	0.003*	-1.49
SLE Number of events	0.80 (1.36)	0.20 (0.45)	0.98	0.334	0.46

*SUVr* = standardized uptake value ratio, *n* = frequency, *M*  = mean, *SD*  = standard deviation, *IQR* = Interquartile range; *t* = t-statistic, *X*_*2*_ = Pearson Chi-square statistic, *Z* = Mann-Whitney U test statistic, *d* = Cohen’s d, *r* = effect size of Mann-Whitney U-Test, *SZ* = Schizophrenia, *DUP* = duration of untreated psychosis, *CDSS* = Calgary Depression Scale for Schizophrenia ; *SUMD* = Scale to Assess Unawareness of Mental Disorder; *SLE* = stressful life events.

* Remained significant after multiple comparison correction.

^a^ Missing data in non-relapsers: DUP (*n* = 41), CDSS (*n* = 41), SUMD total and benefits of antipsychotics (*n* = 35), SUMD existence of mental illness and consequences of mental illness (*n* = 41), SLE (*n* = 41).

^b^ The Fisher’s Exact chi-square statistic was used as expected counts are less than 5.

When both SUVr putamen-to-caudate and *baseline* medication status were entered into a multivariate penalized logistic regression model, SUVr remained a significant predictor of relapse (*p* = 0.037); baseline medication status was also significant in this model (*p* = 0.011). When *follow-up* medication status was entered instead of baseline, SUVr putamen-to-caudate also remained significant (*p* = 0.044), whereas medication status was not (*p* = 0.265). Full regression outputs are provided in Supplementary 2.

### Post hoc power estimation

Given the vast methodological inconsistencies in the relevant literature, including variability in PET parameters, processing pipelines, and outcome metrics, deriving a reliable effect size for an a priori power calculation using traditional tools like G*Power is challenging [24, 25]. Using the observed mean difference in SUVr putamen-to-caudate between relapsers and non-relapsers (0.07) and the standard deviations from Table 3 (0.09 for non-relapsers; 0.03 for relapsers), a post hoc calculation indicates that approximately 27 non-relapsers and 4 relapsers would be required to detect this difference with α = 0.05 and 80% power. Our current sample of 42 non-relapsers and 5 relapsers, therefore, provides reasonable sensitivity to detect the observed effect.

## Discussion

This study examined striatal DSC in remitted psychosis patients and its association with relapse. We found no overall DSC differences between patients and healthy controls. Within the patient group, higher DSC was associated with males, unmarried individuals, having greater impairment in premorbid schizoid-schizotypal traits, better insight, and more severe general psychopathology. Several factors predicted relapse, including lower baseline SUVr putamen-to-caudate ratios, medication discontinuation at baseline, immigrant status, and impaired insight into the benefits of antipsychotic medication. Penalized regression analyses confirmed that baseline SUVr putamen-to-caudate remained a significant predictor of relapse even after accounting for medication effects at both baseline and follow-up, highlighting the potential of DSC as an independent marker of relapse vulnerability in remitted psychosis.

### DSC in remitted patients

No significant differences in DSC were observed between remitted patients and healthy controls, although elevated DSC has been reported in prodromal and acute phases, suggesting phase-specific dopaminergic dysfunction [5, 6]. Studies in remitted patients have produced mixed results: some found no differences compared with healthy controls [9], whereas others reported either elevated [10] or reduced DSC, with reductions particularly observed in treatment-resistant patients compared with responders [26]. In this naturalistic study of minimally symptomatic patients, we observed no overall DSC differences between remitted patients and healthy controls.

Putamen DSC showed greater variability than caudate (putamen-to-cerebellum: *p* = 0.140; caudate-to-cerebellum: *p* = 0.622) (Table 1). Correlations with demographic and clinical variables (Table 2) further indicated that DSC variability is more prominent in the putamen. This aligns with longitudinal PET evidence showing that individuals who transitioned from a prodromal state to psychosis exhibited progressive increases in striatal DSC, peaking in the putamen, while no significant changes were observed in the caudate or other striatal subdivisions [5]. More generally, these findings are also consistent with prior reports emphasizing putamen abnormalities in schizophrenia [4].

Our results also revealed no association between DSC and positive or negative symptom severity, in line with previous studies [10, 26, 27]. Among remitted patients, general psychopathology correlated with DSC, though the association remains complex due to the diverse symptoms, such as anxiety, depression, and preoccupation, encompassed within this subscale. Indeed, an intricate underlying mechanism may exist, necessitating future research to categorize specific symptoms within this subscale.

Higher putamen DSC at baseline was linked to male gender, unmarried status, and greater premorbid schizoid-schizotypal features. In healthy controls, women exhibited higher DSC than men in both the putamen and caudate [28], suggesting potential sex-specific differences in dopaminergic function in psychosis. The association with marital status may reflect age-dependent effects, as married patients were likely older, and DSC generally declines with age [29]. Additionally, the link between premorbid schizoid-schizotypal traits and higher caudate DSC highlights dopaminergic involvement in early psychopathology.

Interestingly, better insight may reduce medication intake, potentially contributing to higher DSC [9]. In contrast to some previous reports, we observed no clear association between antipsychotic use and DSC. Consistent with prior research [30], we found no change in DSC before and after medication, despite symptom improvement. This may reflect the inclusion of remitted patients, in whom antipsychotic effects on DSC are uncertain. Longitudinal studies are needed to clarify the temporal dynamics of antipsychotic effects on DSC.

### Predictors of psychotic relapse

Lower baseline putamen DSC was observed in patients who later relapsed. Although some evidence implicates the associative striatum in dopaminergic dysfunction [31], our findings highlight the putamen, underscoring heterogeneity across striatal subregions in psychosis. The relationship between DSC and relapse appears to vary across illness stages: elevated DSC has been reported before psychosis onset [5], whereas lower DSC in relapsers aligns with findings in treatment-resistant patients [26] and those with limited symptomatic improvement [32]. These findings suggest that reduced dopaminergic capacity in some remitted patients may indicate vulnerability to relapse or poor long-term outcome in psychosis.

While our study measured DSC at a single time point during remission, longitudinal evidence suggests dopaminergic changes may predict relapse. In a prospective PET study, higher baseline striatal DSC predicted shorter time to relapse following antipsychotic discontinuation, despite no differences in D2/3 receptor availability between relapsed, non-relapsed, and healthy controls [9], indicating that relapse may reflect impaired presynaptic dopamine autoregulation rather than DSC normalization. In our naturalistic cohort, which included patients with varied illness durations and medication histories, relapse risk was likely influenced by multiple factors beyond discontinuation.

Differences in remission and relapse definitions may further affect outcomes: prior research [9] used a stringent criterion capturing clinically significant relapses (40%), whereas our more lenient definition aimed to detect even mild symptom exacerbations, yet observed a lower relapse rate (11%). Despite these differences, both studies highlight a role for striatal dopamine synthesis capacity in relapse, although the underlying mechanisms remain unclear.

Antipsychotic effects further complicate the interpretation of striatal DSC. Consistent with prior studies, treatment-resistant patients exhibit lower DSC than responders, reflecting variation in dopaminergic function with treatment response [26, 33]. In our sample, no overall baseline DSC differences were observed between patients on or off medication, likely due to a mix of treatment-responsive and resistant individuals and heterogeneous illness durations and medication histories. Abrupt versus tapered discontinuation may also differentially affect DSC and relapse risk [34]. Furthermore, our findings showed that lower baseline putamen-to-caudate SUVr consistently predicted relapse independent of medication exposure, whereas baseline medication discontinuation, but not follow-up status, also predicted relapse, highlighting the proximal effect of treatment. These findings support baseline DSC as a prognostic biomarker while underscoring the need to account for medication as a potential confounder, though unmeasured variability in the discontinuation process may explain some non-significant results [9, 30, 34, 35].

Beyond biological factors, our study identified additional demographic and clinical predictors of relapse. Consistent with previous longitudinal research [36], we found that a higher proportion of relapsers had discontinued antipsychotics at baseline compared with non-relapsers. Migration also predicted relapse, aligning with earlier studies that reported a link between psychosis onset and immigrant status [37, 38] and potentially reflecting increased exposure to psychosocial stressors [39]. Finally, worse illness insight, specifically unawareness of antipsychotic benefits, was associated with higher relapse risk, aligning with earlier findings [40].

### Methodological consideration

To our knowledge, this is the first study using SUVr as a DSC measurement to predict relapse in remitted psychosis patients. DSC differences in demographic and clinical variables were observed. This suggests that SUVr may be more commonly used among future DSC studies, given its increased feasibility and cost-effectiveness [13, 14]. However, some have indicated that SUVr may lack sensitivity for detecting DSC differences across psychotic disorders, such as schizophrenia and delusional disorders, where only the K influx demonstrated significant findings [12].

Our study attempted to use a better quantification method for SUVr by using the caudate as a reference region to examine DSC in the putamen, as opposed to the conventional use of the cerebellum, which has a lower signal-to-noise ratio. SUVr putamen-to-caudate might be a more representative measurement of DSC, yet replication of the use of this parameter is needed, as it is not commonly employed to examine DSC in psychosis literature.

### Limitations and future directions

This study revealed multiple associations between DSC and clinical outcomes, highlighting the need to examine potential confounding factors, as DSC variations may reflect distinct pathophysiological mechanisms or individual differences. The heterogeneity in schizophrenia’s clinical and neurobiological profiles complicates the development of universally applicable biomarkers and treatments. However, while lower baseline putamen DSC was observed in relapsers, the literature on dopaminergic predictors of relapse is mixed, and our findings are based on a small number of relapsers. Therefore, these results should be considered preliminary and hypothesis-generating, with replication in larger cohorts needed to confirm the prognostic relevance of baseline DSC measures.

Additional limitations include the one-year follow-up with few relapsers, and clinical outcomes and medication status measured only at baseline and follow-up, without longitudinal DSC assessments. Consequently, we cannot rule out that changes in medication or other proximal factors contributed to relapse. Future studies incorporating PET scans before and after antipsychotic discontinuation would clarify dynamic medication effects and disentangle medication influences from underlying neurobiological vulnerability.

Finally, healthy controls were screened only for an absence of a family history of schizophrenia, leaving potential shared genetic risk factors unassessed. Future work should use stricter inclusion criteria, larger samples, longer follow-ups, more rigorous definitions of remission and relapse, and examine demographic and clinical moderators such as sex, age, and antipsychotic profile (e.g., class or polypharmacy).

### Conclusion

This study provides preliminary evidence that lower baseline putamen dopamine synthesis capacity may indicate vulnerability to psychotic relapse among remitted patients. While striatal hyperdopaminergia characterizes schizophrenia at the group level, within-patient variation appears clinically meaningful. Additional demographic and clinical factors, including baseline medication discontinuation, migration status, and impaired insight, further influence relapse risk. These findings highlight the potential prognostic value of DSC measures, while underscoring the need for larger, longitudinal studies that account for medication effects and other confounding factors to clarify the neurobiological mechanisms underlying relapse.

## Supplementary Information

Below is the link to the electronic supplementary material.


Supplementary Material 1



Supplementary Material 2


## Data Availability

The data that support the findings of this study are available from the corresponding author upon reasonable request.
