# Supplementary material for: Dopamine synthesis capacity in patients with remitted psychosis: correlates and one year outcome on relapse
Source: Eur Arch Psychiatry Clin Neurosci. 2026 Jan 6;276(6):2885–94. doi: 10.1007/s00406-025-02185-8 (PMC13525969; doi:10.1007/s00406-025-02185-8)
Supplement: Supplementary file 2 [file 406_2025_2185_MOESM2_ESM.docx]

**SUPPLEMENTARY MATERIAL**

**Supplementary Material 1.** Information of relapsers (*n* = 5).

|  | | | Relapser 1 | Relapser 2 | | Relapser 3 | | Relapser 4 | | Relapser 5 |
| --- | --- | --- | --- | --- | --- | --- | --- | --- | --- | --- |
| Basic Demographic | |  | | |  | |  | |  | |
| Age, year | | | 53 | 26 | | 20 | | 29 | | 38 |
| Gender | | | Female | Female | | Female | | Female | | Female |
| Diagnosis | | | Brief psychotic disorder | Psychosis not otherwise specified | | Schizophrenia | | Psychosis not otherwise specified | | Schizophrenia |
| Clinical characteristics at baseline and relapse | | | | | | | | | | |
| PANSS item and total scores at baseline | | | | | | | | | | |
|  | PANSS P1-Delusion | | 1 | 1 | | 1 | | 1 | | 1 |
|  | PANSS P2-Conceptual disorganization | | 1 | 1 | | 3 | | 1 | | 1 |
|  | PANSS P3- Hallucination | | 1 | 1 | | 1 | | 1 | | 1 |
|  | PANSS P6- Suspiciousness/ Persecution | | 1 | 1 | | 1 | | 1 | | 1 |
|  | PANSS G9-Unusual thought content | | 1 | 1 | | 1 | | 1 | | 1 |
|  | Total PANSS | | 5 | 5 | | 7 | | 5 | | 5 |
| PANSS item sand total scores at relapse | | |  |  | |  | |  | |  |
|  | PANSS P1-Delusion | | 5 | 6 | | 4 | | 5 | | 1 |
|  | PANSS P2-Conceptual disorganization | | 1 | 1 | | 6 | | 1 | | 4 |
|  | PANSS P3- Hallucination | | 5 | 1 | | 3 | | 1 | | 1 |
|  | PANSS P6- Suspiciousness/ Persecution | | 4 | 2 | | 1 | | 4 | | 1 |
|  | PANSS G9-Unusual thought content | | 5 | 1 | | 3 | | 3 | | 1 |
|  | Total PANSS | | 20 | 11 | | 17 | | 14 | | 8 |
| Time to relapse, days | | | 238 | 340 | | 198 | | 334 | | 34 |
| Hospitalization due to relapse | | | No | No | | 62 days | | 14 days | | No |
| Functional characteristics at baseline and relapse | | | | | | | | | | |
| SOFAS at baseline | | | 55 | 51 | | 61 | | 68 | | 78 |
| SOFAS at relapse | | | Missing | Missing | | 31 | | 52 | | 64 |

*Abbreviations*: PANSS = Positive and Negative Syndrome Scale; SOFAS = Social and Occupational Functioning Assessment Scale.

**Supplementary Material 2.** Penalized logistic regression analyses (*n* = 47).

Table 2.1. Penalized logistic regression analysis predicting relapse at 1-year by SUVr Putamen-to-Caudate, controlling for medication status at baseline.

| **Variable** | **B** | **S.E.** | **Wald** | ***p*** | **OR [95% CI]** |
| --- | --- | --- | --- | --- | --- |
| SUVr Putamen-to-Caudate | -14.691 | 7.361 | 4.364 | **0.011*** | 7.13e-02 [2.457e-15, 4.400e-01] |
| Medication Status at Baseline^a^ | -2.641 | 1.074 | 6.470 | **0.037*** | 4.17e-07 [5.425e-03, 5.474e-01] |

*Abbreviations*: B = Regression coefficient (log-odds); S.E. = Standard error of the coefficient; Wald = Wald chi-square statistic; *p* = Significance value (two-tailed); OR [95% CI] = Odds ratio with 95% confidence interval; SUVr = Standardized Uptake Value ratio.

*Note.* Penalized maximum likelihood estimation was used to fit the logistic regression. Firth’s penalized likelihood (logistic regression with bias reduction) was used to reduce small-sample bias.

^a^ Medication status at follow-up: 0=off medication/discontinuation, 1=on medication/continuation.

Table 2.2. Penalized logistic regression analysis predicting relapse at 1-year by SUVr Putamen-to-Caudate, controlling for medication status at follow-up.

| **Variable** | **B** | **S.E.** | **Wald** | ***p*** | **OR [95% CI]** |
| --- | --- | --- | --- | --- | --- |
| SUVr Putamen-to-Caudate | -12.785 | 6.738 | 4.043 | **0.044*** | 2.803e-06 [5.541e-14, 7.487e-01] |
| Medication Status at Follow-up^a^ | -1.071 | 0.959 | 1.244 | 0.265 | 3.425e-01 [3.757e-02, 2.232e+00] |

*Abbreviations*: B = Regression coefficient (log-odds); S.E. = Standard error of the coefficient; Wald = Wald chi-square statistic; *p* = Significance value (two-tailed); OR [95% CI] = Odds ratio with 95% confidence interval; SUVr = Standardized Uptake Value ratio.

*Note.* Penalized maximum likelihood estimation was used to fit the logistic regression. Firth’s penalized likelihood (logistic regression with bias reduction) was used to reduce small-sample bias.

^a^ Medication status at follow-up: 0=off medication/discontinuation, 1=on medication/continuation.
